# Adaptation, acceptability and feasibility of a Short Food Survey to assess the dietary intake of children during attendance at childcare

**DOI:** 10.1017/S136898001900404X

**Published:** 2020-06

**Authors:** Alice Grady, Alison Fielding, Rebecca K Golley, Meghan Finch, Gilly A Hendrie, Tracy Burrows, Kirsty Seward, Christophe Lecathelinais, Sze Lin Yoong

**Affiliations:** 1 School of Medicine and Public Health, University of Newcastle, Callaghan, NSW, Australia; 2 Hunter New England Local Health District, Population Health, Wallsend, NSW, Australia; 3 Hunter Medical Research Institute, Newcastle, NSW, Australia; 4 Priority Research Centre for Health Behaviour, University of Newcastle, Callaghan, NSW, Australia; 5 GP Synergy, Research and Evaluation Unit, Mayfield West, NSW, Australia; 6 College of Nursing and Health Sciences, Flinders University, Adelaide, SA, Australia; 7 Priority Research Centre in Physical Activity and Nutrition, University of Newcastle, Callaghan, NSW, Australia; 8 Health and Biosecurity, Commonwealth Scientific and Industrial Research Organisation, Adelaide, SA, Australia; 9 School of Health Sciences, Faculty of Health and Medicine, University of Newcastle, Callaghan, NSW, Australia

**Keywords:** Nutrition, Child, Food intake, Childcare, Educators, Validity

## Abstract

**Objective:**

To (i) describe the adaptation of the Short Food Survey (SFS) for assessing the dietary intake of children (2–5 years) during attendance at Early Childhood Education and Care (SFS-ECEC); (ii) determine the acceptability and feasibility of the SFS-ECEC; and (iii) compare the SFS-ECEC to direct observations for assessing dietary intake of children in care.

**Design:**

The adapted forty-seven-item SFS-ECEC was completed by childcare educators to capture individual child’s usual intake over the past month. Acceptability and feasibility were assessed via educator self-report and completion rates. Mean servings of food groups consumed in accordance with dietary guidelines reported in the SFS-ECEC were compared to those obtained by a single-day direct observation via visual estimation conducted by trained personnel. Mean differences, intra-class correlations, Bland–Altman plots, percentage agreement and Cohen’s *κ* were examined.

**Setting:**

Early Childhood Education and Care, NSW, Australia.

**Participants:**

Educators and children.

**Results:**

213 (98·61 %) SFS-ECECs were returned. Acceptability was high with 86·54 % of educators reporting the tool as easy to understand. Mean differences in servings of food groups between the SFS-ECEC and direct observation were statistically significantly different for five out of six foods and ranged 0·08–1·07, with intra-class correlations ranging 0·00–0·21. Agreement between the methods in the classification of children meeting or not meeting dietary guidelines ranged 42·78–93·01 %, with Cohen’s *κ* ranging −0·03 to 0·14.

**Conclusions:**

The SFS-ECEC is acceptable and feasible for completion by childcare educators. While tool refinement and further validation is warranted, small mean differences suggest the tool may be useful in estimating group-level intakes.

Early childhood is a critical period for establishing dietary intake patterns for lifelong health and is a key life-stage for obesity prevention initiatives^(1–^ ^3^ ^)^. Early Childhood Education and Care (ECEC) services are gaining increasing recognition as a key setting for nutrition-related primary prevention initiatives^(4)^ with growing childcare service utilisation globally^(3,5–^ ^8^ ^)^. Feasible, accurate and reliable dietary intake measures are essential for assessing the effectiveness of interventions aimed at improving children’s intake in the care setting, as well as for monitoring adherence to childcare-specific dietary guidelines^(2,3,6,9)^.

Measuring dietary intake in young children is complex, where the usual challenges of dietary assessments are compounded by the inability of young children to self-report due to limited communication, knowledge of food types and cognitive functioning^(9,10)^. Parents/guardians are, therefore, often required to act as proxy-reporters, yet their ability to accurately report food consumed is inherently limited for the meals and snacks provided during childcare hours^(9,10)^.

The assessment of dietary intake of children in care settings, to date, has been largely dependent on direct observations or objective data collection of food consumption (e.g. plate–waste measures)^(5)^. While direct observation is considered valid and reliable,^(11)^ this method is costly to conduct, limiting its feasibility as a method for assessing the dietary intake of young children in childcare on a population level^(3,9)^. As such, the practicality of direct observation for epidemiological or evaluation research involving large numbers of children in care settings dispersed across wide geographical areas is limited.

Alternatives to direct observation and collection of objective data exist. There is rapid development in the utilisation of technology-based methods (e.g. photo- or video-recordings) for measuring the dietary intake of children^(2,9)^. However, such methods may be prohibitive due to pragmatic issues, including consent and privacy concerns for young children and childcare services, in addition to costs associated with training staff in the use of technologies, ongoing development and validity concerns. One non-validated educator-reported (as proxy reporters) method of assessing individual child’s intake in care has also been reported in the literature^(12,13)^. However, as this method requires detailed real-time recordings of intake by educators, feasibility limitations are apparent given the burden on time-limited childcare educators and potential impact on service daily schedules and routines.

Short survey-style tools completed by childcare educators provide a potential solution to the challenges faced in measuring individual children’s dietary intake at childcare. Short tools tend to be less than fifty items in length and ask questions about the frequency or servings of foods or food groups, as well as diet quality or dietary habits^(1,14)^. They are quick to administer, with low respondent and administrator burden, and are therefore more feasible for the assessment of dietary intake of children at scale. Reviews of short tools that measure children’s dietary intake have highlighted a lack of validated short dietary intake methods to assess ‘in-care’ intake of children^(1)^.

One short survey tool identified in a recent review with favourable validity and reliability for assessing the dietary intake of young children, compared with other short food questionnaires, is the Short Food Survey (SFS)^(14)^. The SFS is a validated, thirty-eight-item parent-reported measure of individual child’s dietary intake, developed for the Australian population^(15)^. It has been found to perform well in estimating intake across a range of food groups in children aged 4–11 years and for estimating children’s overall adherence to the Australian Dietary Guidelines via an index score^(15)^. On this basis, the SFS has potential utility to be adapted for completion by childcare educators, where maximising accuracy while also maintaining educator acceptability and feasibility are paramount considerations.

Therefore, the aims of this study were to (i) describe the adaptation of the SFS for assessing dietary intake of children (aged 2–5 years) during attendance at ECEC services; (ii) determine the acceptability and feasibility of the SFS-ECEC as reported by a sample of childcare educators; and (iii) compare the SFS-ECEC to direct observations for assessing the daily intake of core food groups (fruit, vegetables, breads and cereals, meat/meat alternatives, dairy/dairy alternatives) and discretionary foods, according to the Australian Guide to Healthy Eating (AGHE), of children attending childcare.

## Methods

### Adaptation of the Short Food Survey for use in Early Childhood Education and Care

An advisory group consisting of dietitians, public health nutritionists, health promotion officers with expertise in the ECEC setting, and the developers of the SFS was formed to identify modifications required to the original SFS for application in the childcare setting using an iterative adaptation and review process (online supplementary material, Supplemental Appendix A for details of adaptations made). In brief, this encompassed modifications to food items assessed/food item examples based on relevance to the ECEC setting; modification of frequency response options to accommodate the assessment of children that attend care for varying numbers of days per week; paper-based survey administration; the addition of portion-size questions to enable the estimation of food group servings; and development of a twelve-page supporting resource flipchart containing images and written examples of serving size portions to assist educators in estimating the food intake of children and categorising foods into groups.

The above process resulted in a forty-seven-item educator-completed dietary assessment tool to capture information on individual child’s (aged 2–5 years) food group intakes and food choices in care over the past month (online supplementary material, Supplemental Appendix B for SFS-ECEC items and responses). Intake and food choice assessment covered the days of the week the child usually attended the childcare service, and the meals the child usually consumed while in care, including breakfast, morning tea, lunch, afternoon tea and late snack. Food groups included the five core food groups described in the AGHE^(16)^, which are fruit, vegetables, breads and cereals, meat/meat alternatives and dairy/dairy alternatives, as well as discretionary choices (i.e. food or beverage items high in saturated fat, added sugars or sodium, and may be energy-dense). The tool also asked about food behaviours, including the consumption of wholegrain choices, reduced-fat dairy, healthy fats and variety (of choices within and between core food groups)^(15)^.

The SFS-ECEC captured data on:

1. Days of the week the child attends care and meals consumed (two items)
2. Frequency of consumption of core AGHE food group items and water (eighteen items)
3. Portion sizes consumed for core AGHE food group items (ten items)
4. Frequency of consumption of discretionary food/beverage items (ten items)
5. Food variety (types of fruit, vegetables, dairy/dairy alternatives, meat/meat alternatives and breads and cereals) (three items)
6. Food choices (wholegrains, fat content of milk, trimmed meat, spread type) (four items)

Pilot-testing of the initial version of the SFS-ECEC was undertaken with a convenience sample of four educators from two childcare services (not included in the current study). Educators were provided verbal and written instructions for the pilot SFS-ECEC and were asked to complete the tool for at least one child each. Feedback was sought, covering tool length, comprehension and feasibility of completion. Feedback supported the feasibility and acceptability of the initial version.

### Acceptability, feasibility and comparison of the Short Food Survey–Early Childhood Education and Care to direct observations

#### Design and setting

An opportunistic cross-sectional study was undertaken to assess SFS-ECEC acceptability, feasibility and to compare two measures of dietary intake of children attending care. The study took place as part of baseline data collection within a randomly selected subset of ECEC services taking part in a randomised controlled trial aiming to improve childcare service compliance with dietary guidelines^(7,17)^.

#### Sample

ECEC services in New South Wales (NSW), Australia, who were clients of a single, specific childcare management software provider, were invited to participate in the randomised controlled trial. To be eligible for this sub-study, services were required to: (i) be open for ≥8 h each weekday; (ii) prepare and provide at least one main meal and two snacks daily to children on-site; and (iii) be able to make menu-planning decisions on-site; and be randomly selected to participate in a 1-day site visit from the research team for the purposes of data collection. Services outsourcing menu planning, not catering for children aged 3–6 years, catering exclusively for special needs children or run by the Department of Education and Communities were excluded.

Educators were required to be present on the day of data collection and allocated to the room in which the highest number of children aged 2–5 years were located. This age range was selected to assess individual child’s intake in care against dietary guidelines for the sector, Caring for Children^(17)^. A pragmatic approach to selecting educators was used to maximise the feasibility of completing individual SFS-ECEC records. Participating educators were required to have familiarity with the typical dietary intake of children undergoing direct observation, as indicated by working regularly (preferably permanently) in the selected room and not being new to the service or room in which dietary observations were taking place.

### Child recruitment

Services provided information statements and consent forms to the parents of potentially eligible children approximately 2 weeks prior to the site visit day. Research assistants present on the day of data collection also approached the parents/guardians during child drop-off to assess eligibility and obtain informed consent for their child to take part.

Children aged 2–5 years, present on the day of data collection, with parent/guardian consent, and with no dietary requirements prohibiting the consumption of foods usually provided were included in the study.

#### Data collection procedures

*Direct observations (reference method).* Children’s dietary intake was measured by research assistants in six randomly selected children per service. Random selection was conducted by a blinded research assistant on the morning of the observation. All participating children were assigned a unique identification number, which was matched against a computer-generated random number table to select the children to be observed. Observations were undertaken using an adapted protocol of the Dietary Observation for Child Care (DOCC)^(18)^, a validated approach for assessing dietary intake in children aged 3–5 years. The DOCC is considered sound for the assessment of dietary intake of children in care^(11)^. As detailed in the protocol for the larger randomised controlled trial^(7)^, prior to service site visits, the research assistants undertook laboratory-based training to ensure accurate identification and estimation of portion sizes of foods and beverages commonly consumed by children in the ECEC setting. Two observers each assessed three children on the day, by documenting the types and portion sizes of food and beverages served to each individual child. At the end of each core meal (morning tea, lunch, afternoon tea), observers recorded the types and portion sizes of foods remaining on the individual child’s plates. Portion sizes were estimated using household measures (e.g. tablespoons), units for foods that are counted in units (e.g. nuggets) or the dimension of foods in centimetres. As per the DOCC protocol, estimated consumption of foods in grams was then calculated^(18,19)^ by a dietitian via Foodworks 9^(20)^. Servings of discretionary foods were calculated by dividing the kilojoules consumed from discretionary foods by a standard serving size (600 kJ) according to AGHE^(16)^. The core food group data were entered into an online programme developed by the research team to classify the number of servings for each food group according to AGHE^(16)^.

*Administration of the SFS-ECEC.* Educators from the room in which the randomly selected children were located completed written questionnaires reporting on the selected children’s characteristics and dietary intake via SFS-ECEC. Educators were asked to complete one SFS-ECEC for each randomly selected child and were asked to complete the SFS-ECECs on the day of site visit or, if this was not possible, to return the completed SFS-ECECs to the research team at a later date via reply paid envelopes provided to the service. Prior to SFS-ECEC completion, the research assistants provided all participating educators with brief training on how to accurately complete the survey, and supporting resources containing example images of foods commonly served in childcare to help with estimating portion sizes. Educators were asked to refer to this supporting resource wherever possible. Immediately following the completion of the SFS-ECEC, educators were asked to complete a brief questionnaire assessing their demographic characteristics, and acceptability and feasibility of the tool.

*Acceptability and feasibility.* Four items were developed by the research team on a four-point Likert scale (ranging from strongly disagree to strongly agree) assessing the clarity (‘I found the questionnaire clear’) and comprehension (‘I found the questionnaire easy to understand’) of the tool, adverse consequences of completion (‘I found the questionnaire distressing’) and usefulness of the supporting resource (‘I found the supporting resource useful for completing the questionnaire’) to assess acceptability^(21)^. These items were completed immediately following the completion of the SFS-ECEC. The feasibility^(21)^ of the SFS-ECEC was also determined by calculating response rate, frequency of missing items, Flesch–Kincaid reading level, self-reported number of SFS-ECECs completed, time to complete, and timeframe from provision of SFS-ECECs to completion.

*Servings of food groups.* Educators reported on children’s frequency of consumption of all six food groups over the past month (thirty-seven items) within the SFS-ECEC for the meals and snacks the child usually consumes in care (two items). Core food groups were also reported by amount in portions (e.g. two portions is equivalent to one serving size consistent with AGHE). Usual frequency and portions of core food groups consumed were then converted into servings per day. As the SFS-ECEC assessed the number of times discretionary foods were consumed rather than servings, a standardised serving size, calculated from the mean kilojoules each time a discretionary food was consumed from direct observations (626 kJ), was applied to the discretionary food frequency of consumption data. As per direct observations, the servings of discretionary foods were then calculated by dividing the kilojoules consumed from discretionary foods by the standard serving size (600 kJ) according to AGHE^(16)^.

*Compliance with childcare guidelines.* According to the NSW dietary guidelines for the sector^(17)^, childcare services are required to provide at least one main meal (e.g. lunch) and two mid-meals or snacks (e.g. morning tea and afternoon tea) over an 8-h period, with these meals providing at least 50 % of the recommended daily servings of each of the AGHE food groups (Table 1). Each food group was considered compliant when the recommended number of servings for that food group according to the NSW dietary guidelines for the sector^(17)^ was observed or reported to be consumed. For direct observations, this reflected the consumption of recommended servings of all food groups on the day of observation; for the SFS-ECEC, this reflected usual daily consumption of recommended servings reported by educators for each child over the past month.

**Table 1:** Australian Guide to Healthy Eating food groups and recommended servings for children while attending care according to the New South Wales Caring for Children Guidelines^(17)^

Food group	Recommended servings
Fruit	1
Vegetables	2
Wholegrain breads and cereals	2
Lean meat and alternatives (e.g. poultry, fish, eggs, tofu, seeds and legumes)	0·75
Dairy (e.g. milk, yoghurt, cheese and non-dairy alternatives)	1
Discretionary foods (e.g. high in kilojoules, saturated fat, added sugars and added salt)	0

#### Other data

Childcare service characteristics, including postcode, number of allocated places and number of educators, were reported by the service-nominated supervisor during service recruitment. Educator characteristics, including qualifications, years worked for the services and days worked each week, and child’s characteristics, including age, sex, days usually attending care and meals and snacks usually consumed in care, were reported by educators.

### Statistical analysis

Statistical analyses were conducted in SAS, version 9.3^(22)^. Descriptive statistics, including means, frequencies and proportions, were used to describe the characteristics of childcare services, educators and children, consumption servings of food groups and compliance with guidelines. Service postcodes, ranked in the bottom 50 % of NSW according to the Socio-Economic Indexes for Areas 2016^(23)^, were classified as being of lower socioeconomic status. Items within the SFS-ECEC without a response were recorded as missing. The following exclusions were made to the dataset: direct observations of children without a corresponding SFS-ECEC and late completers of SFS-ECEC as identified by return of surveys over 1 month post-direct observations. This resulted in a total of two services (encompassing two educators) and twenty-one children (9·72 %) being excluded from the analyses. Further, outliers as identified by food group servings >3 sd from the mean intake according to either direct observations or SFS-ECEC were removed from individual food group analyses (ranging 1–9 per food group).

Multiple methods were employed to compare the estimates of intake derived from the SFS-ECEC to direct observations. For servings of food groups (continuous data), differences between the two methods in mean estimates of intake were assessed using linear mixed model regression with the childcare services as a random effect to account for potential clustering effect. Intra-class correlations (ICC) were calculated to establish group-level association between the two methods^(24)^; Bland–Altman plots to determine the agreement between individual’s absolute values from each method^(25)^; and linear regression analysis for each food group (regression of the average of two methods *v.* their difference) to test if the slope of mean bias was significantly different from zero (group level)^(25)^. Visual examination of histograms determined the mean difference in servings for each food group to be normally distributed. The ability of the SFS-ECEC to categorise children into meeting/not meeting dietary consumption recommendations for each food group (dichotomous data) was also assessed using percentage perfect agreement^(15)^; McNemar’s test to determine the significance of differences between SFS-ECECs and direct observations; and Cohen’s *κ* ^(15)^. Based on the benchmarks suggested by Landis and Koch^(26)^, *κ* measures were classified as follows: poor = <0; slight = 0·00–0·20; fair =0·21–0·40; moderate = 0·41–0·60; substantial = 0·61–0·80; almost perfect = 0·81–1·00.

For the comparison of mean servings of food groups consumed, a total of 195 children across 33 services, assuming an ICC of 0·05 and with an *α* of 0·05, had 80 % power to detect a difference of 0·32 Z units (or 32 % of an sd) between the SFS-ECEC and direct observations.

## Results

A total of 33 services, 52 educators and 195 children were included in the analyses (Table 2). On average, educators were present for almost 5 d of the week, children attended the service 3·42 d out of 5, and over 90 % of children regularly consumed morning tea, lunch and afternoon tea in care, with an additional 15·18 % of children consuming breakfast and 25·65 % consuming a late snack.

**Table 2:** Characteristics of participating childcare services (*n* 33), educators (*n* 52) and children (*n* 195)

Characteristics	*n* or mean	% or sd
Service		
Socioeconomic status		
High	20	60·61
Low	13	39·39
Mean number of allocated childcare places per day	56·82	16·12
Mean number of educators per service	12·58	8·76
Educator		
Number of years employed		
<1 year	15	28·85
1–5 years	22	42·31
>5 years	15	28·85
Mean number of days employed at service per week	4·75	0·58
Highest qualification completed		
Early childhood teaching	27	51·92
Diploma	16	30·77
Certificate III	7	13·46
Other	2	3·85
Child		
Mean age	4·09	0·71
Sex		
Female	91	48·40
Male	97	51·60
Mean number of days in care per week	3·42	1·15
Meals consumed while in care		
Breakfast	29	15·18
Morning tea	173	90·58
Lunch	190	99·48
Afternoon tea	190	99·48
Late snack	49	25·65

### Acceptability, feasibility and comparison of the Short Food Survey–Early Childhood Education and Care to direct observations

The acceptability assessment of the SFS-ECEC determined that 86·54 % of educators agreed that they found the questionnaire clear, 86·54 % agreed that they found the questionnaire easy to understand, 96·15 % found the supporting resource useful for completing the questionnaire, and 19·61 % reported they found the questionnaire distressing. Difficulties in accurately capturing children’s consumption due to variation in child and staff attendance, child self-serving and changing menu cycles were reported by five educators.

Of the 216 SFS-ECECs distributed to educators, 213 (98·61 %) were returned. The frequency of missing responses of any one item ranged 6–11 (3·08–5·64 %). In terms of readability, the Flesch Reading Ease was 64·7 (plain English, easily understood by 13–15-year-olds), and the Flesch-Kincaid Grade Level was 7·9. On average, educators completed 3·61 (sd 1·81) SFS-ECECs, taking an average of 13·59 (sd 8·37) min to complete per child. The questionnaires were completed on average 3 d following direct observation data collection.

### Servings of food groups

The mean difference in the servings of each core food group and discretionary foods between the two measures ranged 0·08–1·07 servings (Table 3), with the SFS-ECEC reporting relatively higher consumption compared to direct observation. Significant differences were found in the mean servings of vegetables, breads and cereals, dairy/dairy alternatives, meat/meat alternatives and discretionary foods between the two methods. The ICC for food group servings was lowest for fruit and meat/meat alternatives (ICC = 0·00). A negative slope of bias was found for three of the six food groups, with linear regression analysis revealing the slope of bias was significantly different from zero for all food groups, with the exception of vegetables.

**Table 3:** Comparison of servings per day for core food groups and discretionary foods calculated using Short Food Survey–Early Childhood Education and Care (SFS-ECEC) and direct observations

	SFS-ECEC		Direct observations		Mean difference	95 % CI^a^	ICC	95 % CI	Slope of bias^b^
	Mean	sd	Mean	sd					
Fruit	0·78	0·48	0·70	0·72	0·08	−0·03, 0·20	0·00	–	−0·72**
Vegetables	0·78	0·50	0·59	0·53	0·19	0·10, 0·29**	0·11	0·03, 0·34	−0·11
Breads and cereals	2·41	1·75	1·35	0·82	1·07	0·83, 1·32**	0·07	0·01, 0·42	1·14**
Dairy/dairy alternatives	1·22	0·75	0·74	0·63	0·48	0·35, 0·61**	0·14	0·05, 0·34	0·26*
Meat/meat alternatives	0·69	0·47	0·32	0·40	0·37	0·29, 0·45**	0·00	–	0·26*
Discretionary foods	0·68	0·49	0·41	0·71	0·28	0·18, 0·39**	0·21	0·11, 0·37	−0·55**

a Linear mixed models regression with random effects accounting for clustering.

b Linear regression analysis (regression of the average of two methods *v.* their difference).

*P < 0·05; **P < 0·001.

**Fig. 1:**
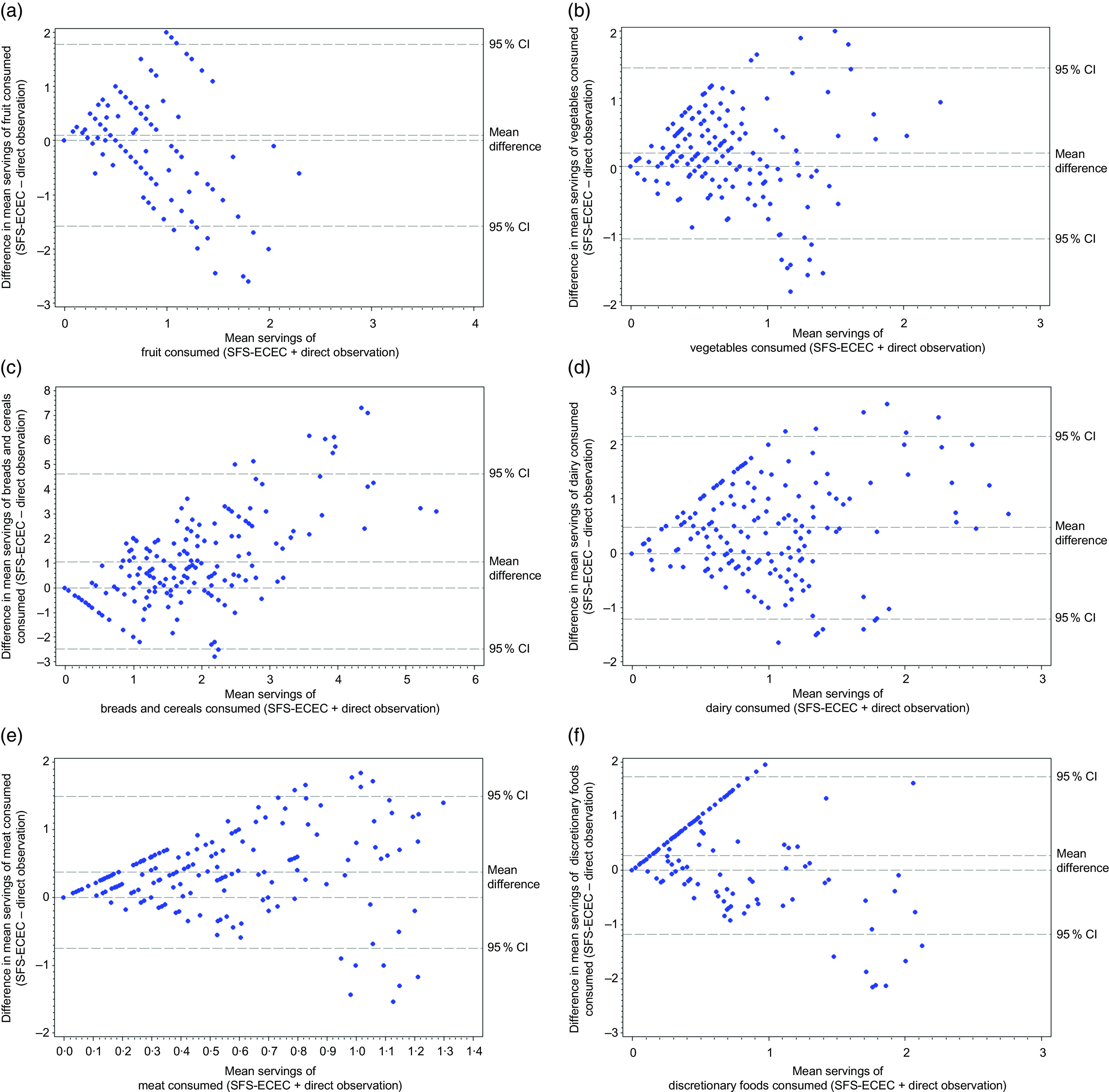
Bland–Altman plots showing agreement between consumption of servings for each food group calculated by Short Food Survey–Early Childhood Education and Care (SFS-ECEC) and direct observations for the following food groups: (a) fruit, (b) vegetables, (c) breads and cereals, (d) dairy/dairy alternatives, (e) meat/meat alternatives and (f) discretionary foods. For each food group, the mean difference in servings between SFS-ECEC and direct observations (*y* axis) was plotted against the mean servings calculated by SFS-ECEC and direct observations (*x* axis), including mean differences and 95 % CIs

A visual examination of Bland–Altman plots (Fig. 1) revealed that with regard to measures of fruit, as the mean of the two measures increased, the greater the underestimation of SFS-ECEC compared to direct observations. For breads and cereals and meat/meat alternatives, as the mean of the two measures increased, the greater the overestimation of SFS-ECEC compared to direct observations; and for vegetables, dairy/dairy alternatives and discretionary foods, as the mean of the two measures increased, the greater the dispersion of SFS-ECEC compared to direct observations.

### Compliance with guidelines

The proportion of children meeting dietary guidelines for the sector while attending care according to the SFS-ECEC ranged 4·76–57·29 %, with discretionary foods showing lowest compliance and dairy/dairy alternatives showing highest compliance (Table 4). Compliance via direct observations ranged 2·08–61·03 %, with lowest compliance for vegetables and highest compliance for discretionary foods. Percentage agreement between SFS-ECEC and direct observations ranged 42·78–93·01 % across all food groups, with significant differences in classifications of meeting or not meeting for all food groups, apart from vegetables (*P* = 0·17). *κ* coefficient values suggest poor agreement between the two measures for fruit and vegetables (*κ* ranging −0·03 to −0·02) and slight agreement for the remaining food groups (*κ* ranging 0·04–0·14). Vegetables had the highest percentage agreement between the two methods, and yet a negative *κ* due to the unequal distribution of agreement between meeting and not meeting guidelines.

**Table 4:** Proportion of children meeting dietary guidelines for the sector, and percentage agreement between SFS-ECEC and direct observations categorised by meeting/not meeting food group recommendations

Food group	SFS-ECEC		Diet observations		*P*-value^a^	Percent perfect agreement	Cohen’s *κ*	95 % CI
	*n*	%	*n*	%				
Fruit	80	41·88	45	23·08	<0·001	53·40	−0·02	−0·15, 0·10
Vegetables	9	4·76	4	2·08	0·17	93·01	−0·03	−0·05, −0·01
Breads and cereals	107	56·02	42	21·88	<0·001	54·26	0·14	0·03, 0·24
Meat/meat alternatives	64	33·68	24	12·50	<0·001	64·17	0·05	−0·07, 0·18
Dairy/dairy alternatives	110	57·29	71	36·60	<0·001	54·98	0·13	0·00, 0·26
Discretionary foods	9	4·64	119	61·03	<0·001	42·78	0·04	0·00, 0·09

a McNemar’s test.

SFS-ECEC, Short Food Survey-Early Childhood Education and Care.

## Discussion

This paper describes the adaptation, acceptability and feasibility of the SFS-ECEC and compares the adapted tool to a single day of direct dietary observations. The SFS-ECEC was based on a previously validated, parent-reported tool, which assesses usual food group consumption and overall diet quality in young children^(15)^. A team of dietitians, public health nutritionists, health promotion officers and SFS tool developers made a number of modifications to the original SFS tool, to enable the assessment of dietary intake of children while attending childcare, to be completed by educators.

The study found high acceptability among childcare staff, as the majority of educators (86·54–96·15 %) reported the SFS-ECEC to be clear, easy to understand and almost all found the flipchart resource helpful to support reporting the dietary intake of children. Surprisingly, 19·61 % of educators reported they found the questionnaire distressing. As this was the only negatively worded item, it is suspected that this finding may indicate response acquiesce. It is also suspected that this distress is related to the overall workload experienced by some educators, with the completion of the tool representing an additional, cognitively challenging task to be completed within a short timeframe. The task of reporting the frequency and portion size for individual children may be difficult for educators in services that encourage children to serve themselves from shared plates or ‘family style’^(27)^. Serving styles, in addition to daily variation in the servings of meals and snacks, the frequency of staff working and the frequency of child attendance per week were noted by five educators as challenges to tool completion. Despite this, the feasibility of tool completion in the childcare setting was high as almost all (98·61 %) of the distributed SFS-ECECs were returned by educators. The average time of completion was 13·6 min per child, and on average, educators completed 3·6 surveys. This is encouraging when compared to direct observations that are conducted over 6 h, with a maximum of three children per observer. Broadly, such findings are reassuring and suggest that the SFS-ECEC is an acceptable and feasible tool to be completed by educators; however, follow-up questions surrounding potential adverse consequences may be warranted.

Overall, the SFS-ECEC reported a higher number of servings for all core food groups and discretionary foods relative to direct observations (mean difference ranging 0·08–1·06 servings across food groups). It is encouraging to note that five of the six food groups were estimated by educators to be within a 0·5 serving of the estimates via direct observation. The largest mean difference between the two methods occurred for breads and cereals. This food group, in particular, may be challenging to estimate due to their inclusion within mixed dishes (e.g. spaghetti bolognaise). Higher reporting of consumption compared to another reference method has been found in other adaptations of the SFS^(28)^. This finding may be due to a number of reasons. Firstly, due to the nature of the questionnaire, spillage or food sharing that is common in meal times with young children may not adequately be captured by the SFS-ECEC. Secondly, the SFS-ECEC captures a greater proportion of a child’s total intake as an additional 15·18–25·65 % of children were reported to consume breakfast and late snack in care. Consumption during this meal and snack was collected in the recording of usual intake via the SFS-ECEC, but not via direct observations, which was limited to morning tea, lunch and afternoon tea. The consumption of breakfast and late snack was unable to be captured via direct observation due to logistical difficulties and resource constraints. Given the additional recording of breakfast and late snack, a higher reported consumption of the breads and cereals food group in SFS-ECEC might be expected, as these foods are commonly served during these meals (e.g. cereals, crackers). Considering these factors, a relatively higher reported consumption by the SFS-ECEC is not unexpected.

The findings reported here are comparable to those reported by the original SFS, which identified that the SFS reported significantly higher servings of core food groups consumed (0·3–1·5 servings) compared to 24-h dietary recalls^(15)^. In contrast to the original parent-completed SFS where discretionary foods were comparatively underestimated, the SFS-ECEC reported higher consumption of discretionary foods. This could reflect differences between educator and parent versions of the SFS where the parents may be more likely to report in a socially desirable manner^(10,28)^.

Low ICCs and wide limits of agreement on the Bland–Altman plots were identified suggesting low levels of agreement between the two methods, and that educator-reported intake of children is likely to vary widely for each individual child. The ICC for fruit and meat/meat alternatives (0·00) suggests there is no agreement, with substantial variation in measures both within and between children. For discretionary foods, an ICC of 0·21 indicates a low level of agreement between the two measures, with slightly less variation within children than between children. The distribution of residual data points in the Bland–Altman plots suggests potential systematic biases in reporting, where the levels of SFS-ECEC reporting are likely higher when mean estimates increase for most food groups. While educators could report children’s consumption of foods any number of times per day, week or month, response options for portion sizes consumed were most commonly limited to half, one or two portions, ultimately placing some parameters on the resulting calculation of food group servings consumed. This may somewhat explain the patterns found within the Bland–Altman plots.

In terms of assessing compliance with guidelines for the childcare sector for each food group, the percentage agreement between methods ranged from 42·78 % for discretionary foods to 93·01 % for vegetables. The high percentage agreement for vegetables was due to the high proportion of children who do not meet the guidelines for recommended servings of vegetables. The original SFS was intended to be used to measure diet quality operationalised as adherence to the Australian Dietary Guidelines^(15)^, rather than a measure of food group intake or dichotomous categorisation of guideline compliance. This reflects validation studies where a diet quality index score may be more comparable to the reference method than individual food group serving assessments^(15,28)^. As such, measures specifically designed to capture food group intake may be better suited for adaptation to the ECEC setting using multiple days of direct observation for comparison.

In considering these findings, it is important to recognise that the SFS-ECEC was designed to capture ‘habitual or usual intake’ over 1 month, while the direct observations were conducted on a single randomly selected day (assuming representation over a usual period). Given the likelihood of variation between usual intake and consumption on single day of observations, individual-level agreement findings (e.g. ICCs and Bland–Altman plots) are not unexpected and should be drawn as preliminary. Further, certain food groups that have a typically less consistent pattern of consumption within the ECEC setting (e.g. discretionary foods) may be more vulnerable to issues arising from the assumption that the 1-d snapshot of intake from direct observations is representative of a child’s ‘usual’ intake. The comparison of one dietary assessment method to another method (of known performance and with conceptually different methodology) is common in the validation of dietary measures as there are no absolute methods of usual intake that are free from error^(9)^. The two methods have important differences giving rise to a comparison that encompasses independent error structures. For example, the methods have different frames of reference (i.e. usual intake over 1 month *v.* consumption on a single day), proportion of in-care intake, and differing cognitive demands. However, based on these differences, the findings of the SFS-ECEC compared to direct observations are in the expected direction. A comparison of the SFS-ECEC to multiple days of direct observation may clarify some of the current findings and would allow for comparison of the diet quality component of the tool. While outlier values were excluded from the analysis for both SFS-ECEC and direct observations, a plausible reporter analysis on direct observation data may provide further insights into the variability found between the two methods.

To our knowledge, the SFS-ECEC is the first tool for which acceptability, feasibility and comparison to a known validated measure has been examined to assess the dietary intake of children in care, and was designed to provide an affordable and pragmatic alternative to existing forms of data collection. The tool was modified to allow completion by educators who are responsible for overseeing meal times of children in care. Although a low relative agreement was observed at the individual level, small mean differences in the consumption of core food groups and discretionary foods were observed, suggesting that the tool may be particularly useful in supporting group-level observations. As such, the tool may represent a feasible and acceptable method for public health or community nutritionists to obtain data regarding food group consumption and food choices of children in order to identify food provision modification required to ensure alignment with sector dietary guidelines.

Modifications and refinement of the tool are recommended to increase its overall acceptability, feasibility and to reduce variation in reporting. While low levels of missing responses were found, the administration of the tool via the web or tablet may reduce both the frequency of missed or unquantifiable items and the time taken to complete the tool. Further, given educators reported a high acceptability of the tool, and yet their estimates of consumption were consistently greater than direct observations, modifications may include additional training for educators (e.g. how to estimate food portion sizes within ‘mixed’ dishes). Amendments to enable the assessment of consumption by individual meals and snacks within SFS-ECEC would likely increase agreement between the two methods. It is noted, however, that such a modification should be weighed against resource intensity and participant burden. Future research to determine the SFS-ECEC’s sensitivity to change is also needed to indicate if this tool can be used to evaluate interventions, in addition to monitoring dietary guideline compliance.

### Strengths and limitations

Strengths of the study include a large sample size (>100 children) adequate for validation studies^(29,30)^; a comprehensively developed tool with input from dietitians, public health nutritionists, health promotion officers with expertise in the childcare setting, child dietary intake measure experts and ECEC service educators; and diligence in selecting educators who would be most appropriate to complete such a measure of child’s usual intake in care.

Despite these strengths, results of the study should be considered in light of the following limitations. Firstly, the SFS-ECEC captures child’s consumption of all meals while in care, whereas dietary observations only occurred for morning tea, lunch and afternoon tea, which is likely to account for some of the relatively higher reporting identified in the SFS-ECEC. As the SFS-ECEC does not assess intake by meal occasion, this was not accounted for in the analysis. Given that certain food groups may be more likely to be served at certain meal occasions (e.g. breads and cereals at breakfast), an assessment of service menus over the reporting period may have provided further insights into the habitual nature of the meals assessed via direct observations. While a higher food group consumption has been reported in the original SFS tool^(15)^ and in other adaptations of the SFS^(28)^, a number of potential factors may contribute to the current findings: certain food groups may be more difficult than others to estimate (e.g. those included in mixed meals); the SFS-ECEC does not capture spillages or food sharing that is common among children; and some food groups may not have consistent provision patterns in care (e.g. discretionary foods). Secondly, the SFS-ECEC and direct observations have different assessment periods of the past month and a single day, respectively, resulting in different error structures between the two methods. Despite this, the use of direct observations for comparison provides a commonly used reference standard to understand the performance of the SFS-ECEC. Thirdly, this study was only conducted in services that provided meals to children (i.e. menu services) and, as such, the utility of the tool for services where foods are brought from home is unknown. Fourthly, while participating educators were required to have familiarity with the typical dietary intake of children subject to the SFS-ECEC, there was no requirement for educators to be present in the room of the child for the last month, potentially reducing the accuracy of educators reporting for the specified time period. Finally, the study was conducted in services within one state in Australia (NSW), and further validation in other jurisdictions would be beneficial.

## Conclusions

In summary, this quantitative dietary assessment tool has been specifically adapted for use in the ECEC setting and for completion by educators. Study findings indicate the tool to be both highly acceptable and feasible for use in the childcare setting. Study findings also indicate low levels of agreement between the SFS-ECEC compared to direct observations; however, small mean differences in the consumption of core food groups and discretionary foods were observed suggesting that the tool may be useful in estimating group-level intakes. Comparison to an alternate reference method or multiple days of direct observations and some refinements to the tool are recommended to increase comparability of results. The study demonstrates that the SFS-ECEC provides an alternate, feasible, acceptable and low-cost method of assessing the consumption of children in Australian ECEC services, overcoming the common feasibility issues that restrict the assessment of child dietary intake in care.

## Supplementary material

For supplementary material accompanying this paper visit https://doi.org/10.1017/S136898001900404X. click here to view supplementary material
