# Supplementary material for: Adaptation, acceptability and feasibility of a Short Food Survey to assess the dietary intake of children during attendance at childcare
Source: Public Health Nutr. 2020 Mar 17;23(9):1484–94. doi: 10.1017/S136898001900404X (PMC7196734; doi:10.1017/S136898001900404X)
Supplement: Supplementary file 1 [file S136898001900404Xsup.zip › S136898001900404Xsup001.pdf]

**Issue identified**	**Adaptation made**	**Original SFS Example**	**SFS-ECEC Example**
Children are enrolled in childcare for differing numbers of days per week. A reference period of one week would not be sufficient to assess usual intake for a child attending only one day per week.	Change in reference period for capturing 'usual' intake	Reference period was specified as over the last week	Reference period was specified as over the last month
Paper-based survey administration (deemed most pragmatic for the current study setting) required flexibility in frequency response options to accommodate: a) children attending care less frequently than 5 days per week, b) foods that may not be served weekly; and c) the potential for an item to not be provided by the service	Change in frequency response options based on food groups being assessed	Response options for all food groups:<br>- ___servings per day<br>- ___servings per week<br>- ___servings per month	Response options for fruit, vegetables, breads and cereals, dairy:<br>- ___times per day<br>- ___times per week<br>- doesn't eat<br><br>Response options for meat and meat alternatives, and discretionary:<br>- ___times per week<br>- ___times per month<br>- doesn't eat/never/not provided by service

Educators may not have knowledge of serving sizes for each food group, but are likely able to provide assessment of frequency of foods.	Change in terminology from ‘servings’ to ‘times’.	“How many servings of <food> does your child usually eat <per time frame>?”	“How many times does the child usually eat <food>?”
Children are often served smaller serving sizes than adults, therefore one time may not be equivalent to one serving	Inclusion of portion size questions to enable more accurate estimation of child servings<br><br>For example, 1 portion = ½ AGHE serve (accounted for in analysis)		“When the child eats <food>, please estimate how much they would usually eat at one time (i.e. at one meal or snack).”<br><br>Response options:<br>- 1/2 portion<br>- 1 portion<br>- 2 portions<br>- doesn’t eat
Educators may not have knowledge of what foods belong in each individual food group	Addition of examples of foods within each food group		Salad vegetables - Includes green leafy and all raw salad vegetables e.g. cherry tomatoes, cucumber, capsicum, lettuce, celery, carrot, beetroot

Certain foods commonly served in childcare services are missing from the original SFS	Items assessing consumption of ‘missing’ foods added		“How many times does the child usually eat plain crackers and crispbreads?”<br><br>“How many times does the child usually eat fish?”
Certain foods included in SFS are not commonly served in childcare	Removed/merged redundant response options	“What type of spread does your child usually have?”<br><br>Response options:<br>- butter<br>- table margarine<br>- unsaturated margarine<br>- doesn’t have spread	“What type of spread does the child usually have?”<br><br>Response options:<br>- butter<br>- margarine<br>- doesn’t have spread
Educators will require training and/or assistance to complete the tool as accurately as possible	12 page supporting resource for educators developed by the research team		See Appendix B
