# Supplementary material for: Adaptation, acceptability and feasibility of a Short Food Survey to assess the dietary intake of children during attendance at childcare
Source: Public Health Nutr. 2020 Mar 17;23(9):1484–94. doi: 10.1017/S136898001900404X (PMC7196734; doi:10.1017/S136898001900404X)
Supplement: Supplementary file 1 [file S136898001900404Xsup.zip › S136898001900404Xsup003.pdf]

Indicator	Question	Response Options
Fruit	How many times does the child usually eat fruit?	Number of times per day / per week / Doesn't eat
	When the child eats fruit, please estimate how much they would usually eat at one.	½ portion / 1 portion / 2 portions / Doesn't eat
	How many times does the child usually drink 100% fruit juice?	Number of times per day / per week / Doesn't eat
Vegetables	How many times does the child usually eat starchy vegetables? *(Includes potato, sweet potato. Does not include deep fried potatoes/chips)*	Number of times per day / per week / Doesn't eat
	When the child eats starchy vegetables, please estimate how much they would usually eat at one time.	½ portion / 1 portion / 2 portions / Doesn't eat
	How many times does the child usually eat salad vegetables? *(Includes green leafy and all raw salad vegetables e.g. cherry tomatoes, cucumber, capsicum, lettuce, celery, carrot, beetroot)*	Number of times per day / per week / Doesn't eat
	When the child eats salad vegetables, please estimate how much they would usually eat at one time.	½ portion / 1 portion / 2 portions / Doesn't eat
	How many times does the child usually eat cooked vegetables? *(Includes all cooked vegetables e.g. broccoli, spinach, carrots, pumpkin, green beans; dried/canned beans, peas or lentils. Includes fresh, canned and frozen varieties. Remember to include vegetables served in mixed dishes e.g. zucchini slice)*	Number of times per day / per week / Doesn't eat
	When the child eats cooked vegetables, please estimate how much they would usually eat at one time.	½ portion / 1 portion / 2 portions / Doesn't eat
Breads and Cereals	How many times does the child usually eat bread? *(Includes bread, rolls, flat bread, crumpets, English muffins and scones)*	Number of times per day / per week / Doesn't eat

	How many times does the child usually eat pasta, rice, noodles or other cooked cereals? *(Includes barley, buckwheat, couscous, semolina, polenta, bulgar or quinoa)*	Number of times per day / per week / Doesn’t eat
	How many times does the child usually eat plain crackers and crispbreads? *(Includes for example rice crackers, rice/corn cakes/thins, cruskits and wheat-cracker biscuits. Does not include savoury shape-style biscuits or flaky-style crackers e.g. Jatz, SAOs*)	Number of times per day / per week / Doesn’t eat
	How many times does the child usually eat breakfast cereals? *(Includes wheat cereal flakes/biscuits, muesli, porridge. Does not include high sugar/high salt/low fibre cereals such as chocolate/plain rice puffs, sweet puffed cereals, corn flakes*)	Number of times per day / per week / Doesn’t eat or not provided by service
	When the child eats breads or cereals, please estimate how much they would usually eat at one time.	½ portion / 1 portion / 2 portions / Doesn’t eat
Meat and Meat Alternatives	How many times does the child usually eat red meats? *(This includes lean beef, lamb, veal. Remember to include red meats in mixed dishes e.g. bolognaise)*	Number of times per week / per month / Doesn’t eat
	How many times does the child usually eat white meats? *(This includes cooked lean chicken, turkey, pork. Remember to include white meats served in mixed dishes e.g. chicken and vegetable soup)*	Number of times per week / per month / Doesn’t eat
	How many times does the child usually eat meat products? (*This includes sausages, frankfurts, devon, fritz, bacon, salami, ham, hot dogs, hamburgers, chicken nuggets*)	Number of times per week / per month / Doesn’t eat
	How many times does the child usually eat fish? (*This includes cooked fish fillets, canned tuna. Remember to include fish served in mixed dishes e.g. salmon patties)*	Number of times per week / per month / Doesn’t eat

	When the child eats red meat/ white meat/fish, please estimate how much they would usually eat at one time.	½ portion / 1 portion / 2 portions / Doesn’t eat
	How many times does the child usually eat legumes or other meat alternatives? (*This includes canned beans/legumes, chick peas, split peas. Remember to include meat alternatives served in mixed dishes e.g. bean salad*)	Number of times per week / per month / Doesn’t eat
	How many times does the child usually eat eggs?	Number of times per week / per month / Doesn’t eat or not provided by service
	When the child eats legumes, other meat alternatives or eggs, please estimate how much they would usually eat at one time.	½ portion / 1 portion / 2 portions / Doesn’t eat
Dairy Products	How many times does the child usually drink milk? *(Remember to include milk used on breakfast cereals)*	Number of times per day / per week / Doesn’t drink
	When the child drinks milk, please estimate how much they would usually drink at one time.	½ portion / 1 portion / 2 portions / Doesn’t drink
	How many times does the child usually eat cheese?	Number of times per day / per week / Doesn’t eat
	When the child eats cheese, please estimate how much they would usually eat at one time.	½ portion / 1 portion / 2 portions / Doesn’t eat
	How many times does the child usually eat yoghurt or custard?	Number of times per day / per week / Doesn’t eat
	When the child eats yoghurt or custard, please estimate how much they would usually eat at one time.	½ portion / 1 portion / 2 portions / Doesn’t eat
Beverages	How many times does the child usually have soft drink, cordial or sports drinks?	Number of times per day / per week/ Never or not provided by service

	How many times does the child usually drink water per day? (*This includes water from drink bottles, bubblers and cups at meal/snack times and throughout the day)*	Number of times per day / Doesn't drink water
Discretionary Foods	How many times does the child usually have meals or snacks from takeaway food stores?	Number of times per week / per month/ Never or not provided by service
	How many times does the child usually eat deep fried potato gems/chips/hash browns, hot chips/French fries, wedges, fried potatoes?	Number of times per week / per month/ Never or not provided by service
	How many times does the child usually eat savoury snacks such as crisps, pretzels, plain/flavoured savoury shape biscuits or flaky style crackers (e.g. Shapes, SAOs)?	Number of times per week / per month/ Never or not provided by service
	How many times does the child usually have sweet biscuits/cakes/buns/muffins/slices/doughnuts?	Number of times per week / per month/ Never or not provided by service
	How many times does the child usually eat savoury pastries such as meat pies, sausage rolls and pasties?	Number of times per week / per month/ Never or not provided by service
	How many times does the child usually eat snack type bars such as muesli and cereal bars?	Number of times per week / per month/ Never or not provided by service
	How many times does the child usually have chocolate or lollies?	Number of times per week / per month/ Never or not provided by service
	How many times does the child usually have ice cream or ice-blocks?	Number of times per week / per month/ Never or not provided by service
Variety	Think back to the two most recent days the child has attended the service. How many different types of the fruit did he/she eat?	Total number of different types

	Think back to the two most recent days the child has attended the service. How many different types of the vegetables did he/she eat?	Total number of different types
	Think back to the two most recent days the child has attended the service. How many different types of the red or orange vegetables did he/she eat?	Number of different types
	Think back to the two most recent days the child has attended the service. How many different types of the green vegetables did he/she eat?	Number of different types
	Think back to the two most recent days the child has attended the service. How many different types of the dairy foods did he/she eat?	Number of different types
	Think back to the most recent week the child has attended the service. Which of the following meat or alternatives has the child eaten over this past week? (Select all that apply)	Beef / lamb / Pork / Veal / Chicken / Turkey / Process meats (e.g. bacon, devon, ham) / Fish / Eggs / Nuts / Tofu or other meat alternatives / Legumes or beans (e.g. bakes beans, chickpeas) / Lentils / None of the above / Other please specify
	Think back to the most recent day that the child has attended the service. Which of the following breads and cereal foods did he/she eat? (Select all that apply)	Bread (brown, flat bread, mixed grain, pita bread, rolls, rye, white, wholegrain) / Breakfast cereals other than muesli or porridge / Oats, muesli, porridge / Pasta, noodles or couscous / Pearly barley or other grains / Polenta, taco shells or tortilla / Rice (brown or white) / None of the above / Other please specify

Food Choices	Based on what the child usually eats when attending your service, how often is the bread that he/she eats wholegrain/wholemeal?	Always / Usually / Sometimes / Never / Doesn't eat bread
	What type of milk does the child usually have?	Whole / Reduced fat / Skim / Soy / Reduced-fat soy / Doesn't have milk / Other please specify
	How often does the child have lean meat or meat that was trimmed before cooking? i.e. all/some of the visible fat cut off the meat before cooking	Always / Usually / Sometimes / Never / Doesn't eat meat
	What type of spread does the child usually have?	Butter / Margarine / Doesn't have spread
